# Maxillary Sinus Augmentation Using Ceramic Alloplastic Granules or Paste: An Experimental Study in Rabbits

**DOI:** 10.3390/dj9060065

**Published:** 2021-06-03

**Authors:** Michael Medeiros Costa, Daniele Botticelli, Ofer Moses, Yuki Omori, Shigeo Fujiwara, Erick Ricardo Silva, Samuel Porfirio Xavier

**Affiliations:** 1 Department of Oral and Maxillofacial Surgery and Periodontology, Faculty of Dentistry of Ribeirão Preto, University of São Paulo, São Paulo 14040-904, Brazil; michael.bmf@usp.br (M.M.C.); erickricardo.rp@gmail.com (E.R.S.); spx@forp.usp.br (S.P.X.); 2 ARDEC Academy, 47923 Rimini, Italy; daniele.botticelli@gmail.com (D.B.); info@omori-dent.com (Y.O.); flyingtaxidriver12@gmail.com (S.F.); 3 Department of Periodontology and Dental Implantology, School of Dental Medicine, Tel Aviv University, Tel Aviv 69978, Israel; 4 Department of Oral Implantology, Osaka Dental University, Osaka 573-1144, Japan

**Keywords:** animal study, sinus floor elevation, bone healing, histomorphometry, alloplastic, collagen membrane

## Abstract

Background: Due to the lack of data comparing the biological behavior of two formulations, granules and paste, of alloplastic graft from microtomographic and histomorphometric points of view, the aim of the present experiment was to compare the histomorphometric and microtomographic healing of two formulations, i.e., granules (MR sites) or paste (MR-inject sites) of an alloplastic graft composed of a combination of beta-tricalcium phosphate and hydroxyapatite used for maxillary sinus lifting. Methods: A sinus lifting procedure was carried out bilaterally in 20 rabbits, and the elevated space was filled with either paste or granules of an alloplastic material. A collagen membrane was placed on the antrostomy and the animals were euthanized after 2 or 10 weeks, 10 animals each group. Microtomographic and histological analyses were performed. Results: Higher proportions of new bone formation were found at the MR, compared to the MR-inject sites both after 2 weeks (2.65 ± 2.89% vs. 0.08 ± 0.12%; *p* < 0.01) and 10 weeks of healing (34.20 ± 13.86 vs. 23.28 ± 10.35%; *p* = 0.022). Conclusions: It was concluded that new bone formation was faster in the MR sites, compared to the MR-inject. However, a longer time of healing should be allowed to make final conclusions about the efficiency in bone formation of the paste formulation of the biomaterial used in the present study.

## 1. Introduction

Implantology plays an important role in the oral rehabilitation of toothless patients, being considered an alternative to conventional fixed and removable prostheses. This therapeutic option makes it possible to restore patients’ function and aesthetics in an effective and predictable manner. The resorption of the alveolar process in the posterior region of the maxilla and the dimensions and extension of the maxillary sinuses can hinder the installation of osseointegrated implants in this region [1]. Increasing the volume of the distal segments of the maxilla applying a sinus floor elevation has become a frequent clinical approach. This surgical method results in a high success rate [2]. However, accurate CBCT [3] or magnetic resonance imaging [4] evaluations should be performed before the surgical procedure to allow a proper treatment plan and avoid possible juxta- and postoperative complications [5,6].

Despite the increasing use of biomaterials, autogenous bone grafting is still considered the gold standard for bone grafting procedures [7,8,9,10] and sinus floor augmentation [11]. However, the use of autogenous bone in bone augmentation surgeries is associated with a series of disadvantages, such as limited bone availability, increased surgical morbidity, longer surgical time, increased risk of paresthesia and infections, increased financial cost, and need for hospitalization in cases of extra-oral donor site [12,13,14]. Moreover, an important reduction in the dimensions of the elevated space should be expected [15,16].

To counteract the disadvantages of using autogenous bone, research using xenogenous grafts has gained strength and has been widely used in maxillary sinus lift surgeries [11]. Xenografts have been shown to allow a high degree of osteointegration of mini-implants installed after sinus floor elevation [17,18]. However, the possibility of disease induction in the host through prions contained within xenogenous grafts redirected the focus of current research on the use of synthetic (alloplastic) materials [19,20].

In this context, synthetic ceramics based on calcium phosphate have been widely disseminated and used, mainly hydroxyapatite (HA), β-tricalcium phosphate (β-TCP), or a mixture of both (HA/β-TCP). These biomaterials come in the form of blocks, granules, or injectable pastes and showed high biocompatibility and osteoconductive capacity [21,22].

A high concentration of β-TCP in a bone substitute material is characterized by greater graft reabsorption during the incorporation period, with a consequent decrease in bone volume. However, a lower concentration of this same component can decrease the potential for new bone formation. The rate of absorption of β-TCP, compared to HA, is faster, allowing the creation of spaces that favor the filling by osteoprogenitor cells, as well as accelerating the bone regeneration process [23,24,25,26].

Biphasic calcium phosphate ceramics grafts have shown predictable and satisfactory results when used in maxillary sinus lift surgeries [27]. One of the main advantages of biphasic calcium phosphate ceramics, compared to other bone substitutes, is its low absorption rate, which gives the graft volumetric stability and long-term mechanical resistance, that is, it acts as a support for cell migration and deposition of bone matrix [22,28,29].

The combination HA/β-TCP has been extensively studied in preclinical and clinical trials, and the combination of these two biomaterials in a single product allows for a better three-dimensional volumetric graft maintenance due to the presence of HA and rapid bone neoformation due to β-TCP resorption when compared to its isolated forms [24,25,30,31]. In a randomized controlled clinical trial [32], sinus floor augmentation was performed with either 60HA/40β-TCP or a deproteinized bovine bone mineral. Higher amounts of new bone were found in the sinus augmented with alloplastic graft, compared to xenograft. Moreover, while new bone is formed on the periphery of the DBBM granules [33], it grows also inside the residues of granules of 60HA/40β-TCP concomitantly to graft resorption [34,35].

Pastes made of allograft or xenograft have been also used for sinus floor augmentation [36,37]. However, to our knowledge, there is still a need to yield further lines of evidence on the healing when biomaterials are used in pastes. Hence, the aim of the present experiment was to compare the histomorphometric and microtomographic healing of two formulations (i.e., paste and granules) of an alloplastic graft composed of a combination of beta-tricalcium phosphate and hydroxyapatite used for maxillary sinus lifting.

## 2. Materials and Methods

### 2.1. Ethical Statement

The experimental protocol was approved by the Ethical Committee of the Faculty of Dentistry in Ribeirão Preto of the University of São Paulo on 9 January 2019 (CEUA N 2018.1.842.58.2). The study was reported according to the ARRIVE guidelines. The guidelines for animal care adopted in Brazil were strictly followed.

### 2.2. Animal Sample

In this prospective, randomized, split-mouth study, 20 adult male New Zealand White rabbits, weighing approximately 3.5–4.0 kg and 5–6 months old, were used. The animals were divided into 2 groups of 10 rabbits each, according to the experimental periods of 2 and 10 weeks.

The experimental model of the split-mouth type eliminates interference between individuals from the same group and allows the use of a reduced number of animals to guarantee the sample’s representativeness. The result of the sample calculation was obtained by the G * Power 3.1 software for this study (considering alpha = 0.05, test power = 0.8, a correlation between measures of 0.5), similar to what was performed in previous studies. Thus, for this configuration, 10 animals per group were considered sufficient to disclose statistically significant differences in new bone percentage between groups.

### 2.3. Biomaterials

Maxresorb^®^ (Botiss Biomaterials, Zossen, Germany) is a bone graft substitute of alloplastic origin, commercially available in the form of granules or paste. The granular formulation consisted of a combination of hydroxyapatite (HA) and beta-tricalcium-phosphate (β-TCP), in the proportion 60:40, respectively, being composed of macropores of 200–800 μm and porosity of 80%. The injectable paste formulation (Maxresorb^®^ Inject) consisted of a controlled absorption paste containing nanoparticles of HA and small granules of HA and β-TCP (also in the proportion 60:40), dispersed in a water-based gel. The nanoparticles of HA (size 15–50 nm) provided an extensive surface area for cellular interactions.

The antrostomies were covered with a resorbable collagen membrane (Bio-Gide^®^, Geistlich, Wolhusen, Switzerland).

### 2.4. Randomization and Allocation Concealment

The randomization between Maxresorb (MR) and Maxresorb-Inject (MR-Inject) groups was performed electronically (http://www.randomization.com accessed on 25 January 2019) by a collaborator who was not involved in the handling of animals and/or in surgical procedures (SPX). The treatment to be performed was notified to the surgeon immediately after the antrostomies of the maxillary sinuses were performed bilaterally.

No information for the histological and microtomographic analyses was provided to the examiner (MMC) regarding the allocation of the two different conformations of grafts and evaluation periods. However, the presence of remnants of gel + nanoparticles allowed the discrimination of the treatments.

### 2.5. Surgical Procedures

All surgeries were performed by an expert maxillofacial surgeon (ERS). Anesthesia was performed using acepromazine (1.0 mg/kg, sc, Acepran^®^, Vetnil, Louveira, São Paulo, Brazil) and with a xylazine solution (3.0 mg/kg im, Anasedan^®^, Sespo Indústria e Comércio LTDA, Paulínia, São Paulo, Brazil) and ketamine (50.0 mg/Kg im, Dopaser^®^, Sespo Indústria e Comércio LTDA, Paulínia, São Paulo, Brazil). After anesthesia, the animals received a prophylactic dose of oxytetracycline dihydrate (40 mg/kg, im, Terramicina LA^®^, Zoetis Indústria e Produtos Veteratórios, Campinas, São Paulo, Brazil), 1% ketoprofen (Ketofen, Bimeda-Mogivet Farmacêutica SA, 3.0 mg/kg, im; Monte-Mor, São Paulo, Brazil) and tramadol hydrochloride (1.0 mg/kg; sc; Halexlstar; Goiânia, Goiás, Brazil). Anti-inflammatory and analgesic medications were maintained for the first three postoperative days.

After trichotomy, antisepsis of the nasal dorsum was performed by topical application of 1% aqueous polyvinyl–pyrrolidone iodine solution (Riodeíne^®^ Tincture, Rioquímica, São José do Rio Preto, São Paulo, Brazil). The incision site was infiltrated with 1/2 tube of 2% mepivacaine and epinephrine at 1:100,000 (Mepiadre^®^, Nova DFL, Rio de Janeiro, Rio de Janeiro, Brazil) and a sterile transparent adhesive (3M, Sumaré, São Paulo, Brazil) was applied for surgical wound protection. An incision of approximately 2.0 cm in length was made along the midline of the nasal dorsum, followed by tissue divulsion and periosteal detachment. The nasal bone was exposed, and the nasal-incisal suture and the nasofrontal suture were identified.

**Figure 1:**
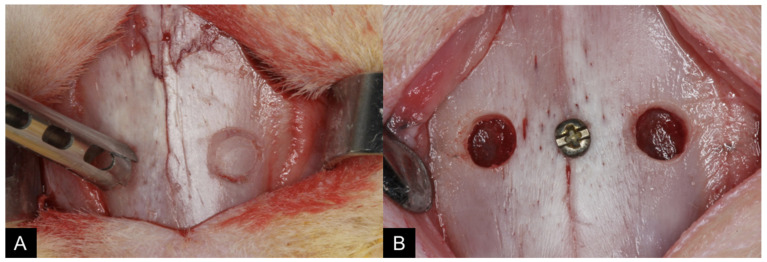
View of the clinical procedures at the experimental region: (**A**) A 3.5–4 mm antrostomy was prepared on both sides, laterally to the nasal-incisal suture, and anteriorly to the nasal-frontal suture using a trephine, and (**B**) the bone windows were removed.

With the aid of a 3.0 mm diameter trephine and a spherical diamond drill (Neodent, Curitiba, Paraná, Brazil), under constant irrigation with sterile saline solution, antrostomies were performed on both sides of the nasal dorsum (Figure 1A,B), according to the protocol already carried out and published previously [38].

**Figure 2:**
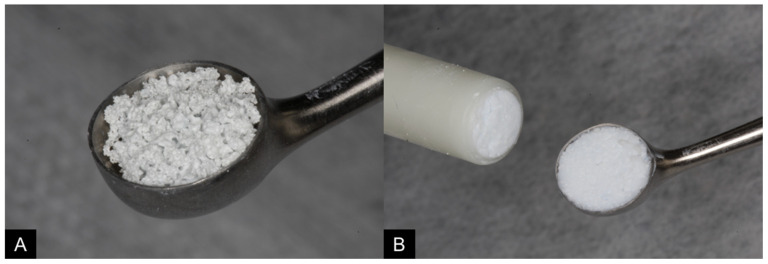
Similar volumes of synthetic alloplastic bone substitute in granules ((**A**); Maxresorb^®^) or paste ((**B**); Maxresorb^®^ inject) were used.

After the elevation of the sinus membrane, similar volumes previously fractionated (~100 mm^3^) of synthetic alloplastic bone substitute in granule (Maxresorb^®^; Figure 2A) or paste (Maxresorb^®^ inject; Figure 2B) formulation were randomly introduced in the maxillary sinuses with the aid of amalgam carrier clamps and maxillary sinus curettes.

**Figure 3:**
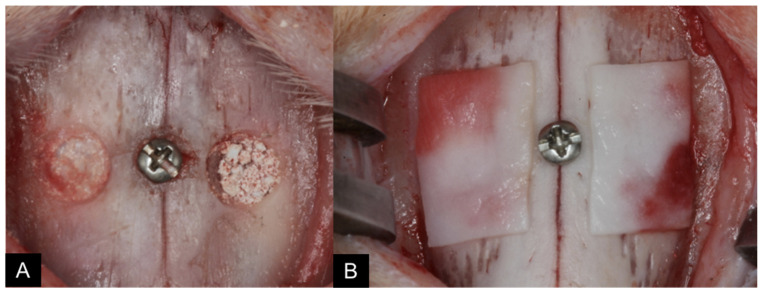
As a central reference for the grafts, a metallic screw was placed in the nasal-incisal suture, at the level of the center of both windows (**A**). An absorbable porcine collagen membrane ((**B**); Bio-Gide^®^, Geistlich, Wolhusen, Switzerland) was used to cover antrostomies bilaterally.

As a central reference for the grafts, a metallic screw was placed in the nasal-incisal suture, at the level of the center of both windows (Figure 3A). An absorbable porcine collagen membrane (Bio-Gide^®^, Geistlich, Wolhusen, Switzerland) was used to cover antrostomies bilaterally (Figure 3B).

For suture, Vicryl ^®^ 4-0 (Ethicon^®^, Johnson & Johnson^®^, São José dos Campos, São Paulo, Brazil) was used in the periosteum and muscle planes and Nylon 4-0 (Ethicon^®^, Johnson & Johnson^®^, São José dos Campos, São Paulo, Brazil) on the skin.

### 2.6. Maintenance Care

After arriving at the vivarium of the Faculty of Dentistry of Ribeirão Preto of the University of São Paulo (FORP/USP), the animals went through a supervised quarantine period to gain weight and check for possible behavioral changes.

All animals were kept in individual steel cages (1.0 animal/4500 cm^2^), under appropriate veterinary care and nutritional support. The animals were housed in a room that had split air-conditioning (21 °C), an exhaust fan (27 to 34 air changes/hour), and automatic lighting control (12 h light–dark cycle). The animals received feed and filtered water ad libitum.

An animal monitoring protocol was carried out during the entire experimental period, through attention to basic biological functions, signs of behavior in relation to postoperative pain, and evaluation of operative wounds for bleeding, suture dehiscence, and/or signs of infection.

### 2.7. Euthanasia

After periods of 2 and 10 weeks (*n* = 10 animals/period), rabbits were euthanized by the anesthetic overdose method with xylazine (3.0 mg/kg im, Anasedan^®^, Sespo Indústria e Comércio LTDA, Paulínia, São Paulo, Brazil) and ketamine (50.0 mg/kg im, Dopaser^®^, Sespo Indústria e Comércio LTDA, Paulínia, São Paulo, Brazil); then, the animals were placed in a closed transparent acrylic box containing gas carbon dioxide (CO_2_).

A block of the nasal region of each animal, containing the two filled maxillary sinuses, was carefully collected and immediately submerged in a 10% formaldehyde buffered solution.

### 2.8. MicroCT Evaluations

After fixation, the pieces were subjected to an X-ray beam scan on the high-resolution microtomograph (SkyScan 1172^®^, Bruker, Kontich, Belgium) at the Ribeirão Preto Dental School-USP.

The following scanning parameters were used: exposure of 570 ms per movement, a voltage of 89 kV, current of 112 μA, isotropic resolution of 8.70 μm, 360° rotation around the vertical axis with 0.2° rotation step, an average of 4 frames, using an Al + Cu filter.

The next step consisted of reconstructing the images using the NRecon^®^ 1.6.10 software (Bruker, Kontich, Belgium), followed by the following parameters: reduction of ring-shaped artifacts (Ring Artifact Correction) in the amount of 12, hardening Beam Hardening of 34%, and smoothing (Smoothing) of 2. The reconstructed images were repositioned three-dimensionally using the DataViewer^®^ 1.5.4.6 software (Bruker, Kontich, Belgium), in which the volume of interest was determined (VOI) of the specimens.

After obtaining the VOI, the CTAn^®^ 1.17.7.2 + software (Bruker, Kontich, Belgium) was used to perform the measurements by interpolating the regions of interest (ROI), marking only the grafted areas in both maxillary sinuses of the rabbits.

Then, the images were binarized, and the grayscale was determined at two different thresholds. For the identification of mineralized tissue, the limits of this scale were set at values from 70 to 100, and for the identification of residual bone graft structures, it was 100–255, as published in a previous study [39].

The volumes of mineralized tissue and graft obtained by the three-dimensional morphometric analysis of the total interpolation of the regions of interest (ROI) were evaluated. A single calibrated examiner performed all analyses (MMC).

### 2.9. Histological Preparation

After microtomographic analysis, the specimens were taken to the FORP-USP Histology Laboratory to begin histological preparation. Initially, the pieces were washed under running water for the complete removal of the fixing agent. Then, they were dehydrated in a series of alcohols of increasing concentrations, changed every three days, under constant agitation.

Subsequently, the specimens were embedded in resin (LR WhiteTM Hard Grid, London Resin Co Ltd., Berkshire, UK) for impregnation and later polymerization in an oven at 60 °C. After the polymerization phase was completed, each block was cut in a coronal plane, in the center of the elevated area, using the metallic screw positioned in the nasal-incisal suture as a reference.

Two sections of approximately 100–150 µm were prepared using precision cutting/grinding equipment (Exakt, Apparatebau, Norderstedt, Germany) and ground until histological slides with an approximate thickness of 50–60 µm were obtained. The slides were stained with either toluidine blue or Stevenel’s blue and alizarin red, as performed in previous studies by our research group [38,40].

#### Calibration for Morpho-Histometric Evaluations

The analyses were made by a single examiner (MMC), after training with an experienced professional. The measurements within each area were taken twice, and an average was calculated. An intraexaminer Kappa test was applied to recognize histological structures, reaching a K > 0.90.

### 2.10. Histomorphometric Evaluations

A Leica DMLB optical microscope (Leica, Wetzlar, Germany), equipped with a digital camera (Digital Sight DS-2Mv, Nikon Corporation, Tokyo, Japan) connected to a computer, was used to obtain the photos of the slides.

**Figure 4:**
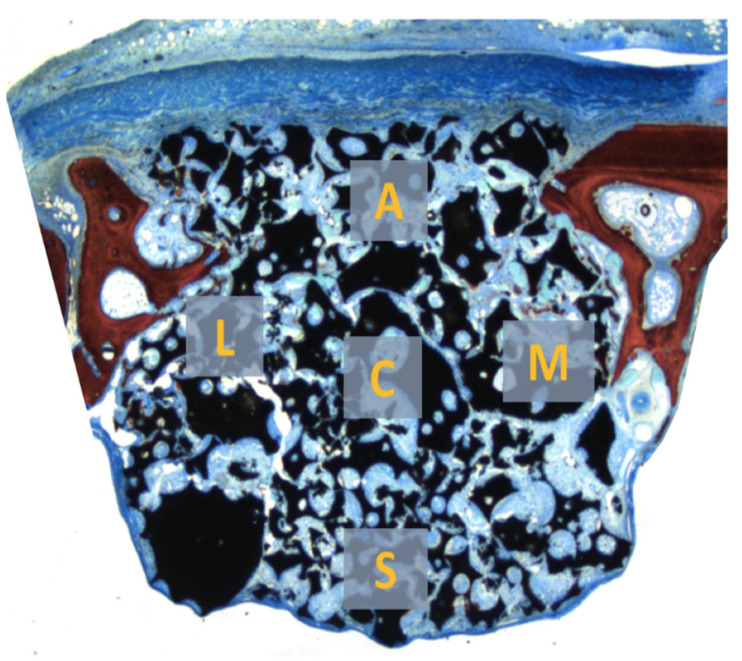
Five distinct areas from the grafted site were determined and used for analysis as follows: (A) the antrostomy region; the areas close to the (M) medial bone wall and (L) lateral bone wall; (C) the central area of the graft; (S) sub-Schneiderian region, subjacent the sinus mucosa.

Five distinct areas from the grafted site were determined and used for analysis as follows: (A) the antrostomy region; the areas close to the (M) medial bone wall and (L) lateral bone wall; (C) the central area of the graft; (S) sub-Schneiderian region, subjacent the sinus mucosa (Figure 4).

The software Image J 1.50i (National Institutes of Health, Bethesda, MD, USA) was used for the measurements. A grid of 80 squares was superimposed on the images of the histological slides, and the points of intersection between the squares were used as a reference to account for the structures present in each of the five areas, at 100× magnification.

Histomorphometric measurements were calculated separately for each area. Mean and standard deviation values were calculated to assess bone neoformation (primary variable) and graft resorption. The evaluated structures included the percentages of new mineralized bone, graft granules’ remnants, paste and granular residues in the paste, vessels, inflammatory infiltrate, osteoblasts, and osteoclasts’ zones. In addition, the percentage of new bone in the regions of the bone walls (lateral wall + medial wall) and the central region of the graft was averaged. Then, a descriptive analysis of the histological findings in each of the experimental periods was performed.

### 2.11. Data Analysis

The primary variable was the new bone percentage, and the secondary variable was the percentage of bone graft remnants, both as evaluated in the histological analyses. The data obtained were tabulated and submitted to statistical analysis using the IBM SPSS Statistic software (IBM Inc., Chicago, CA, USA). The results were expressed as mean ± standard deviation. The differences between MR and MR-inject sites were evaluated applying the Wilcoxon test, while differences between the two periods were evaluated using the Mann–Whitney U test. The level of significance was 5% (*p* < 0.05).

## 3. Results

### 3.1. Microtomographic Evaluation

**Figure 5:**
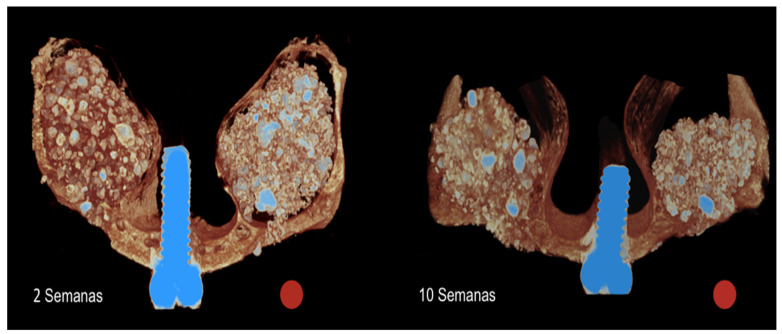
Tridimensional microtomographic images representing the healing after 2 and 10 weeks at the MR (red circles) and MR-inject groups.

Similar amounts of both biomaterials were placed after the elevation of the sinus mucosa (~100 mm^3^). After 2 weeks, the total volume of the elevated region (Figure 5) was 104.8 ± 20.9 mm^3^ and 133.7 ± 34.6 mm^3^ at the MR and MR-inject groups, respectively (*p* < 0.005). After 10 weeks, the respective volumes decreased to 74.1 ± 16.0 mm^3^ and 88.3 ± 38.6 mm^3^ (*p* = 0.153). The amount of new bone increased between 2 and 10 weeks from 17.5 ± 3.2 mm^3^ to 23.2 ± 1.6 mm^3^, in the MR group (*p* < 0.05), and from 20.7 ± 9.0 mm^3^ to 25.2 ± 6.4 mm^3^, in the MR-inject group. No statistically significant differences were disclosed between MR and MR-inject groups.

The amount of residual graft increased from 18.3 ± 2.8 mm^3^ to 23.5 ± 6.3 mm^3^, in the MR group, and from 9.2 ± 2.6 mm^3^ to 16.3 ± 4.3 mm^3^, in the MR-inject group, between 2 and 10 weeks (*p* < 0.05). In the analysis between groups, a greater amount of residual graft was observed in the granule group after 2 and 10 weeks of repair (*p* < 0.05).

### 3.2. Descriptive Histological Evaluation

**Figure 6:**
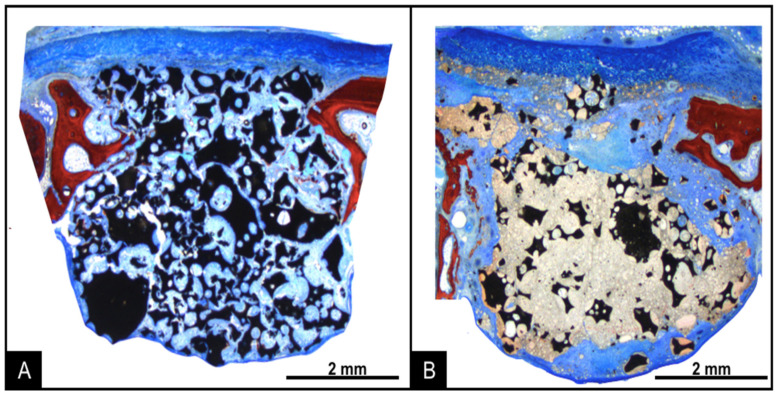
Photomicrographs of ground sections representing the healing after 2 weeks at the MR (**A**) and MR-inject (**B**) groups. Stevenel’s blue and alizarin red stain. 16× magnification.

After 2 weeks of healing, the raised maxillary sinus area had a uniform dome contour lined by the sinus mucosa (Figure 6).

**Figure 7:**
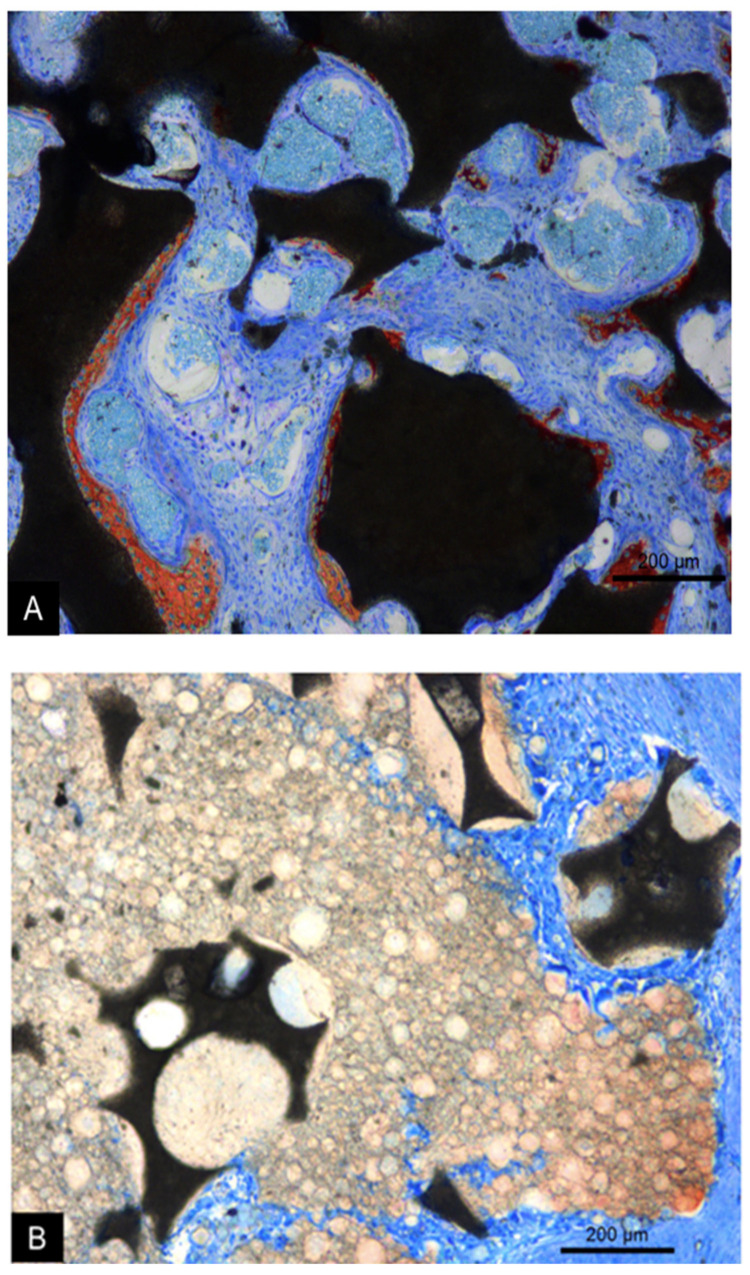
Photomicrographs of ground sections representing the healing after 2 weeks at the MR (**A**) and MR-inject (**B**) groups. In the MR group (**A**), new bone was formed on the granules, separated by vascularized connective. In the MR-inject group (**B**), smaller diameter particles were embedded into water-based gel containing nanoparticles. Note osteoclast on the surface of the water-based gel. Stevenel’s blue and alizarin red stain. 100× magnification.

The collagen membrane was still visible in the region of antrostomies in both groups. In the MR group, small amounts of new bone were found in the regions close to the bone walls. On the surface of the biomaterial particles, a thin layer of immature bone and osteoblasts was observed (Figure 7A). A large amount of biomaterial particles of varying size and irregular shape was observed in the grafted area, surrounded by soft tissues. In the MR-inject group, little bone was observed. Granules of biomaterial were present, included into residues of the water-based gel-containing nanoparticles. Soft tissue surrounded the gel remnants and penetrated within its mass. Osteoclasts were observed on the surface (Figure 7B).

**Figure 8:**
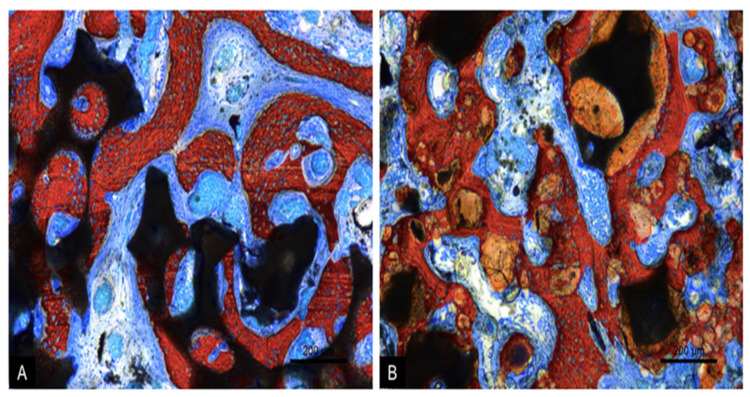
Photomicrographs of ground sections representing the healing after 10 weeks at the MR (**A**) and MR-inject (**B**) groups. In the MR group (**A**), bridges of new bone interconnecting the residual granules of the graft were observed. Vascularized connective tissue was interposed among the granules. In the MR-inject group (**B**), a significant increase in new bone and a decreased amount of gel components were noted. New bone bridges were interconnecting the small and the large granules. Vascularized connective tissues were filling the space among the granules. The spaces between the particles and the paste were filled with vascularized connective tissue. Stevenel’s blue and alizarin red stain. 100× magnification.

After 10 weeks of healing, the uniform dome contour of the grafted region was maintained. The collagen membranes at the antrostomies were reabsorbed, with few remnants, and higher amounts of new bone were observed (Figure 8A,B).

In the MR group, new bone was found formed in all regions, laying of the surface of remnants of graft granules and, in some instances, interpenetrating the biomaterial. Vascularized soft tissues were filling the spaces among new bone and graft remnants (Figure 8A).

In the MR-inject group, new bone was found surrounding the graft remnants. However, the gel remnants were, in several cases, interpenetrated by new bone. Higher amounts of new bone were found close to the bone walls, compared to the central regions. The graft particles appeared not to be resorbed completely, compared to the 2-week period, while the proportion of gel markedly decreased (Figure 8B). The antrostomies of both sites were partly corticalized.

### 3.3. Histomorphometric Evaluation

In the histomorphometric analyses, new bone was found increasing between 2 weeks (Table 1) and 10 weeks of healing (Table 2) in both groups, from 2.65 ± 2.89% to 34.20 ± 13.86% in the MR sites (*p* < 0.01), and from 0.08 ± 0.12% to 23.28 ± 10.35% in the MR-inject sites (*p* < 0.01). The difference in the new bone between MR and MR-inject groups was statistically significant at both 2- (*p* < 0.01) and 10-weeks (*p* = 0.022) of healing.

In the MR group, the highest percentages of new bone were found close to the sinus bone walls (~4–5%; Table 3). However, after 10 weeks, similar proportions of new bone were found in all regions, ranging between 32 and 37% (Table 4). In the MR-inject group, very little bone was found after 2 weeks of healing in all regions (Table 3). After 10 weeks, similar amounts of new bone, compared to the MR group, were found in the regions close to the bone walls. However, the regions far away from the parent bone presented lower proportions of new bone, being the differences with the MR group statistically significant (Table 4).

The graft in the MR group decreased between 2- and 10-weeks from 52.05 ± 9.01% to 37.78 ± 14.57%, while in the MR-inject, only the gel presented a reduction in percentages from 59.53 ± 12.78% to 25.98 ± 17.86%, while the proportion of granules remained stable (Table 1 and Table 2).

## 4. Discussion

The present work aimed to compare the histomorphometric and microtomographic healing of two formulations (i.e., paste and granules) of an alloplastic graft composed of a combination of beta-tricalcium phosphate and hydroxyapatite used for maxillary sinus lifting.

In the study, 2 and 10 weeks of healing were used. The 2-week period was selected because it was shown in previous studies that in this period, useful information could be gathered about the sources of bone formation and about the influence of the two fillers on the response of the various tissues analyzed [35,40]. A 10-week period was applied to allow a longer time of healing, compared to that used in other similar studies, that did not show complete healing of the elevated subantral space. In those studies, however, different biomaterials from those used in the present study were applied [33,38].

After 2 weeks of healing, little bone was formed, being the proportion higher in the MR sites compared with the MR-inject sites, mostly located in the regions close to the bone walls. After 10 weeks of healing, new bone increased to 34%, in the MR sites, and to 23%, in the MR-inject sites, being the difference between the two groups statistically significant. The difference was mainly due to the higher content of new bone in the regions of the sinus far from the bone walls, i.e., central, sub-Schneiderian, and near-window, registered in the MR sites, compared to the MR-inject sites. That outcome showed a faster new bone formation rate in the MR group, compared to the MR-inject group. This outcome was complemented by a lower rate of graft resorption at the MR-inject, compared to the MR sites.

The biomaterial in granules used in the present study was also applied in other clinical [30,41] and experimental [42,43] studies. In a clinical study [30], biopsies were collected six months after sinus floor elevation performed either with 100% beta-tricalcium phosphate (β-TCP) or a biphasic 60% hydroxyapatite (HA) and 40% ß-TCP. New bone was found in proportions of 36.2% and 38.4%, respectively. Optimal results have been also reported in animal studies when used to fill both artificial femoral condylar defects in rats and alveoli immediately after extraction in dogs [42,43].

In the present study, one sinus was filled with granules of biomaterial, while in the contralateral, a paste of the same biomaterial was used. Biomaterial in paste formulation has been used in experimental [44,45] and clinical [36,37] studies.

In an experiment in rabbits [44], defects were created in the maxilla and filled with prehydrated and collagenated porcine bone particles with or without the addition of collagen gel. Different periods of healing were evaluated, and no differences were observed in new bone formation between the two different formulations of biomaterial. In a multicenter randomized control trial [37], 37 patients underwent maxillary sinus floor augmentation with deproteinized bovine bone mineral or prehydrated and collagenated porcine bone particles with the addition of collagen gel. Biopsies were collected for histological examination after 6 months of healing. Similar proportions of bone were found in both groups (~37%).

Nevertheless, in the present study, differences in new bone formation were observed between the two biomaterials used after both 2 and 10 weeks of healing. The histological data after 10 weeks performed in the different regions of the elevated space showed that similar proportions of new bone were observed in the regions close to the bone walls in both groups. However, while in the MR sites, similar amounts of new bone were found in all regions evaluated, lower amounts of new bone were observed in the regions of the MR-inject sites located far from the bone walls, namely, in the sub-mucosa, middle- and near-window regions. It has been shown that new bone is formed mainly from the sinus bone walls [33,38,46] as well as from the bone window displaced inward the sinus cavity or repositioned on the antrostomy [40,47]. The Schneiderian mucosa has been shown to have an osteogenic role [48,49,50]. However, in clinical situations, this potential has been disputed due to the transient edema at which is exposed the sinus mucosa during the first period of healing [5,51,52,53,54] and to the lack of differences in bone formation [38,55,56] or volume of the elevated space [53] when the sinus mucosa was partially excluded from the elevated space with the placement of a collagen membrane. Moreover, no new bone formation from the sinus mucosa was observed during the first month of healing in an experiment in monkeys [57,58].

The paste of the MR-inject included granules of different dimensions. It has been shown that the dimensions of the granules did not influence new bone formation and bone apposition at implant installed simultaneously to sinus lift [59,60].

In the present study, the antrostomies appeared to be close with newly formed bone, partly corticalized. The closure of the antrostomy might be influenced by several factors such as its dimensions [52,61], the device used for the preparation [62], the use of a collagen membrane on the antrostomy [54], the repositioning of the bone window [40], or the placement of autogenous bone particles on the antrostomy [63]. When a collagen membrane was used, the antrostomy presented lower bone proportions, compared to the regenerated bone inside the elevated subantral space [64].

The microtomographic evaluation proposed in this study aimed to investigate three-dimensionally the volumetric variations of the maxillary sinuses grafted with Maxresorb^®^ in granules or paste, through the analysis of the total graft volume, amount of newly formed mineralized tissue, and remaining graft in two experimental times (i.e., 2 and 10 weeks). After 2 weeks, the total volume increased in the MR-inject groups, compared to the amount of xenograft used at the surgery. This might be related to the absorption of liquid consequent to the postsurgical edema. After 10 weeks, the total volume decreased by about 12% in the MR group, and 26% in the MR-inject group, compared to the amount used at the surgery. There was difficulty in determining the grayscale (threshold) that accurately differentiated new bone from the remaining graft. This same limitation regarding microtomographic analysis of grafts in the maxillary sinus of rabbits has recently been described by other authors [39,65]. In the present study, for the purpose of standardization and comparison, two grayscales (threshold) were determined, one for assessing mineralized tissue (threshold 70–100, mm^3^) and another for residual bone graft (threshold 100–255, mm^3^), as proposed by another experimental study [39].

The total volume of the graft showed a reduction of 29.5%, for the granule group, and 33.9%, for the paste group, after 10 weeks. This result may be associated with the presence of a greater amount of graft remaining in the granule group, mainly because the water-based gel has been reabsorbed.

As limitations of the present study should be mentioned the experimental model used that presents a faster rate of healing [66] and the time of healing that should have been longer to allow complete resorption of the biomaterial at the MR-inject sites that might have allowed a further new bone apposition.

## 5. Conclusions

The outcomes from the present experiment allowed the conclusion that new bone formation was faster in the MR sites, compared to the MR-inject. However, a longer time of healing should be allowed to make final conclusions about the efficiency in bone formation of the paste formulation of the biomaterial used in the present study.

## Figures and Tables

**Table 1:** Healing after 2 weeks: soft tissue percentages within the elevated sinusal space. Mean values ± standard deviation. Granules = remnants of granules of HA and β-TCP; Gel = remnants of gel-containing nanoparticles of HA and β-TCP. Graft = Granules + Gel at the MR-inject sites.

	New Bone	Soft Tissue	Granules	Gel	Graft Total	Vessels	Infiltrate	Osteoblasts	Osteoclasts
MR	2.65 ± 2.89	43.80 ± 8.15	52.05 ± 9.01	NA	52.05 ± 9.01	0.68 ± 1.37	0.15 ± 0.39	0.25 ± 0.31	0.43 ± 0.50
MR-Inject	0.08 ± 0.12	19.60 ± 15.52	20.20 ± 4.72	59.53 ± 12.78	79.73 ± 16.13	0.05 ± 0.11	0.55 ± 1.21	0.00 ± 0.00	0.00 ± 0.00
*p*-values MR vs. MR-inject	<0.01	<0.01	<0.01		<0.01	0.065	0.465	0.041	0.027

**Table 2:** Healing after 10 weeks: soft tissue percentages within the elevated sinusal space. Mean values ± standard deviation. Granules = remnants of granules of HA and β-TCP; gel = remnants of gel-containing nanoparticles of HA and β-TCP. Graft = Granules + Gel at the MR-inject sites.

	New Bone	Soft Tissue	Granules	Gel	Total Graft	Vessels	Infiltrate	Osteoblasts	Osteoclasts
MR	34.20 ± 13.86	26.08 ± 5.95	37.78 ± 14.57	NA	37.78 ± 14.57	1.43 ± 1.59	0.18 ± 0.37	0.20 ± 0.33	0.15 ± 0.27
MR-Inject	23.28 ± 10.35	26.58 ± 10.41	22.65 ± 5.39	25.98 ± 17.86	48.63 ± 20.48	1.03 ± 1.20	0.30 ± 0.63	0.13 ± 0.32	0.08 ± 0.12
*p*-values MR vs. MR-inject	0.022	0.646	<0.01	NA	0.028	0.056	0.892	0.257	0.480
*p*-values 2 vs. 10 weeks MR	<0.01	<0.01	0.028	NA	NA	0.369	1.000	0.647	0.158
*p*-values 2 vs. 10 weeks MR-inject	<0.01	0.174	0.325	<0.01	<0.01	0.076	0.804	0.147	0.067

**Table 3:** Healing after 2 weeks: new bone percentages within the various regions evaluated within the elevated sinusal space. Mean values ± standard deviation.

	Medial	Lateral	Schneiderian	Central	Near Window
MR	4.38 ± 6.33	4.63 ± 3.44	2.50 ± 3.86	1.50 ± 3.53	0.25 ± 0.53
MR-Inject	0.25 ± 0.53	0.00 ± 0.00	0.13 ± 0.40	0.00 ± 0.00	0.00 ± 0.00
*p*-values MR vs. MR-inject	0.018	<0.01	0.066	0.109	0.157

**Table 4:** Healing after 10 weeks: new bone percentages within the various regions evaluated within the elevated sinusal space. Mean values ± standard deviation.

	Medial	Lateral	Schneiderian	Central	Near Window
MR	36.00 ± 15.57	32.50 ± 18.16	32.13 ± 14.25	33.50 ± 14.43	36.88 ± 14.29
MR-Inject	31.38 ± 13.84	29.63 ± 12.67	17.50 ± 14.94	16.00 ± 13.08	21.88 ± 14.91
*p*-values MR vs. MR-inject	0.333	0.385	0.032	<0.01	0.012

## Data Availability

The data are available on reasonable request.
